# Is it Possible to Recover Cardiac Functions After Total Knee Arthroplasty?

**DOI:** 10.2174/1874325001812010261

**Published:** 2018-07-19

**Authors:** Aydın Arslan, Bilal Çuglan, Bülent Özkurt, Ali Utkan, Mehmet Fatih Korkmaz, Tuba Tülay Koca, Resit Sevimli

**Affiliations:** 1 Department of Orthopaedics and Traumatology, Elite İstanbul Medical Center,Istanbul Gelisim University,Istanbul,Turkey; 2 Department of Cardiology, Silivri Medical Park Hospital,Bahcesehir University,Istanbul,Turkey; 3 Department of Orthopaedics and Traumatology, Ankara Numune Research and Training Hospital, SBU,Ankara,Turkey; 4 Department of Orthopaedics and Traumatology,Turgut Ozal Medical Center,Malatya,Turkey

## Abstract

**Background:**

Patients suffering from knee osteoarthritis lead a less active life than their healthy peers. It is well known that insufficient physical activity is the most common cause of chronic diseases. However, there is not enough research to enlighten the effect of increased functional capacity on cardiac functions after Total Knee Arthroplasty (TKA). This study aimed to investigate whether the orthopedic surgeons can predict that the patients will be healthier after TKA in terms of cardiac functions or not?

**Methods:**

109 patients who underwent TKA were prospectively followed for one year. The Western Ontario and McMaster Universities Osteoarthritis Index (WOMAC) and short form 36 (SF-36) surveys, BMI measures, average step count per day, the six-minute walking test (6MWT), the Five-Times-Sit-to-Stand Test (FTSST) and Doppler echocardiography were performed both in the preoperative and postoperative period.

**Results:**

After TKA, there was a substantial improvement in terms of WOMAC and SF36 survey scores. The average step count increased from 2199.6±690.8 steps/day to 4124.3±1638.8 steps/day. 6MWT and FTSST improved significantly as well. The average brisk walking time was 174.23±95.11 minutes/week. The means of early and late mitral inflow velocity ratios (E/A and Em/Am ratios) increased from 0.71±0.12 to 0.77±0.13 and from 0.66±0.13 to 0.76± 0.15 at the first year follow-up visit, respectively (p<0.001).

**Conclusion:**

In the first year, objective physical capacity measures increased together with the expected improvements in disease-specific and generic measures. After TKA, left ventricular diastolic functions may be considered to have recovered in the light of the healing signs *via* echocardiography.

## 1 INTRODUCTION

Knee osteoarthritis is the most common type of osteoarthritis characterized by pain and decreased health-related quality of life values [1, 2]. Consequently, patients suffering from knee arthritis lead a less active life than their healthy peers. It is also a well-known fact that people at a relatively older age are under higher risk of morbidity and mortality [2, 3]. Different aspects of physical functioning are monitored using the following procedures: Self-report questionnaires associated with Health-Related Quality Of Life (HRQOL) such as disease-specific and generic measures, functional capacity measurements such as 6MWT and FTSST, step count by pedometer and actual physical activity measurements using activity monitor [4, 5]. Although the patient’s perception of physical function is improved after TKA, the findings of an increase in actual physical activity and decrease in sedentary behavior have been debated [6, 7]. Whether actual physical activity level increases after TKA or not is an important issue. However, there is an increase in functional capacity measured by objective testing procedures as well [5, 7]. Irrespective of the arguments about actual physical activity status after TKA, the effects of the objectively measured increase of functional capacity and physical activity to general health should be considered as another important point. With aging and inactivity, as a consequence of structural and functional changes, left ventricular diastolic dysfunction occurs [8]. There are some evidence demonstrating left ventricular diastolic functional changes associated with aging decrease with physical activity [9-11]. But there is not enough study about the consequence of left ventricular diastolic dysfunction after TKA. The present study aimed to investigate whether the orthopedic surgeons can predict that the patients will be healthier after TKA in terms of cardiac functions or not?

## 2 MATERIALS AND METHODS

This study was approved by Malatya Ethical Committee (Institutional Review Board of Turgut Ozal Medical Center, Inonu University, Malayta/Turkey, Protocol Number: 2014/178). Patients who underwent TKA were followed prospectively for at least one year after the operation. On the 45^th^ day of follow-up, patients were advised to walk at a brisk rate for 30 minutes every day in fifteen-minute sessions in the morning and the afternoon. The targeted walking time was 150 minutes per week. Patients with the following conditions were excluded from the study as these conditions prevent them from brisk walking: neurological diseases, lumber disc herniation, advanced osteoarthritis or disorders of hip, contralateral knee and ankle joints, systolic heart failure, coronary arterial diseases, advanced heart valve disease, congenital heart disease, hypertrophic cardiomyopathy, atrial fibrillation, pulmonary hypertension, chronic obstructive pulmonary disease. Patients with postoperative complications, such as pulmonary thromboembolism were also excluded as they caused immobility and prevented patients from taking part in the walking program. Patients who cannot use the pedometer properly, and those who have undergone any major surgical procedures that may prevent mobility after TKA were also excluded from the study. 205 patients were hospitalized for TKA. 36 patients with bilaterally advanced knee osteoarthritis were excluded since they could not adapt to the brisk walking program due to excessive pain on the other knee. 2 patients did not want to participate in the study. 13 patients had no ability to perform the walking program due to other disorders. Thus, preoperative evaluations were carried out on 154 patients. 6 operations were cancelled. Then, a brisk walking program was planned for 148 patients. 1 patient was hospitalized in an intensive care unit on the second postoperative day due to pulmonary thromboembolism. This patient was excluded due to a special rehabilitation program requirement. Deep vein thrombosis was diagnosed in 1 patient on the third postoperative day. This patient was treated successfully and included in the study. 148 patients were given a brisk walking program. 1 patient underwent drainage and debridement in the third month due to persistent pain and high levels of erythrocyte sedimentation rate and C-reactive protein. Patients who could not properly use a pedometer and record times and those who did not come to follow-ups regularly and could not complete the brisk walking program were excluded. The patient inclusion flow diagram is presented in Fig. (**1**). Finally, 109 patients were followed at least one year and completed the brisk walking program and all evaluations.

Infection prophylaxis was performed using 1gr cefazolin one hour before the operation. Ice therapy and the ankle pump exercise were started immediately after the operation. Isotonic and isometric knee exercises were started on the first postoperative day. Then patients were encouraged to sit in bed and bedside and mobilized with walker immediately. Patients were discharged after the forth postoperative day. Low molecular weight heparin was used for thromboembolism prophylaxis. 40 mg/0.4 ml enoxaparin sodium (Clexane^®^4000 anti-Xa/ 0.4ml pre-filled syringes, Sanofi Aventis) was given subcutaneously 12 hours after the operation and continued once a day for 3 weeks. Morbid obese patients were given 60 mg/0.6 ml enoxaparin sodium (Clexane^®^ 6000 anti-Xa/ 0.6 ml pre-filled syringes, Sanofi Aventis). WOMAC [12] as a disease-specific and SF-36 survey [13] as a generic HRQOL, BMI, the average step count per day, the six-minute walk test (6MWT), the Five-Times-Sit-To-Stand Test (FTSST) and Doppler echocardiography were performed both in the preoperative and postoperative period. Pedometer (SW-200 Yamax Digiwalker) was used to count the steps. For staged operations, preoperative evaluations were performed before second TKA operation. WOMAC and SF36 evaluations, BMI measure, 6MWT and FTSST were performed one week before the operation, and repeated in a follow-up session one year after the operation. Average step count per day was measured during last week preoperatively and the average of one week before 6^th^, 9^th^, 12^th^ months follow-ups postoperatively. The patients were asked to record both the number of daily steps and brisk walking time one week before the follow-up visits. Brisk walking time was estimated by taking into account the averages recorded one week before the 6th, 9th, 12th-month follow-ups postoperatively. Preoperative and postoperative echocardiographs were performed by the same two cardiologists.

Preoperative WOMAC score, SF36 survey, BMI, average step counts per day and echocardiography parameters were compared with postoperative values. 6MWT is a valid testing procedure, evaluating the exercise capacity of the elderly, rather than their daily activities. Patients were asked to walk on a 30-meter track with their pace. They were allowed to rest when they felt tired [14]. At FTSST, patient sits with arms folded across chest and with their back against a 16 inches standard height chair. Then, patient is asked loudly to stand up and sit again five times as quickly as possible. The duration till 5^th^ standing position is recorded. The five-times-sit-to-stand test is reported as a valid test in terms of dynamic balance and functional mobility in older adults [15]. All echocardiographic examinations were performed with the 4-mhz transducer of Vivid 7 pro (Vivid 7 pro, GE Vingmed, Milwauikee, Wisconsin, USA). Interpretation of echocardiographic examinations was performed by two cardiologists blinded to ECG measurements of the study population. Two-dimensional and pulsed Doppler measurements were performed according to the criteria of the American society of echocardiography. The following two-dimensional parameters were measured: Left Ventricular End-Diastolic Diameter (LVEDD, mm) Left Ventricular End Systolic Diameter (LVESD, mm), Left Ventricular Ejection Fraction (LVEF, %), diameters of Left Atrium (LA). The LVEF was estimated using Simpson’s rule. Statistical analyses were performed using SPSS Statistics for Windows, version 21.0 (IBM Corp., Armonk, NY). Kolmogorov Smirnov test was used to check whether the data were normally distributed or not. If the data was normally distributed, paired samples t-test was used. Two related sample t-tests were used in the case of non-normally distributed data. It was supposed that α equaled 0.05 and 1-β (power) equaled 0.80 for the power analyses. A p-value <0.05 was considered statistically significant.

The mean age was 66.75±6.25 (54-85) years (Table **1** for patients’ characteristics). The BMI decreased from 31.65±3.95 (23.1-41.5) kg/m2 to 30.97±3.9 (21.23-40.6) kg/m2 (p <0.001). There was a statistically significant increase in terms of WOMAC scores and SF36 survey scores with the exception of a subscale which is role limitation due to emotional problems (Tables **2** and **3**). Average step count per day increased from 2199.6±690.8 (453-3800) steps/day to 4124.3±1638.8 (1700-12096) steps/day (p <0.001). 6MWT and FTSST scores significantly improved (p <0.001) (Table **4**).

Average brisk walking time was 24.88±13.58 (10-63) minutes/day and 174.23± 95.11 (70-441) minutes/week. The means of E/A and Em/Am values increased from 0.71±0.12 (0.40-0.97) to 0.77±0.13 (0.48-1.26) and from 0.66±0.13 (0.33-1.22) to 0.76±0.15 (0.41-1.42), respectively, at first-year follow-up visit (p<0.001). There were no significant differences in diameters of LV dimensions (LVEDD, LVESD, IVSd), diameters of LA and LVEF between the preoperative and postoperative first-year follow-up visit (Table **5**).

## 4 DISCUSSION

Aging is a major risk factor that increases the incidence of osteoarthritis [16]. It is obvious that left ventricular diastolic functional changes take place with aging as well [8, 17]. The present study was designed since there is not enough research to enlighten the effect of increased functional capacity on cardiac functions after TKA. Contrary to the traditional view that associates the surgical success with the implant survival, 20% of the patients who have undergone the TKA complain functional inabilities and/or residual pain [2]. In this context, the evaluation of HRQOL derived from patients becomes more important [1, 2]. In the present study, HRQOL evaluation was performed *via* WOMAC as a disease-specific measure and SF36 survey as a generic measure. The objective functional capacity evaluation was performed *via* average step count per day, 6MWT and FTSST. Left ventricular diastolic functions were evaluated by Doppler echocardiography. There was a statistically significant increase both in HRQOL and objective functional capacity measures. There was a statistically significant increase in early and E/A and Em/Am ratios.

It has been reported in a number of previous studies that although there was an increase in HRQOL values, there was no measurable increase in actual physical activity levels [6, 7, 18]. Harding *et al.* [6] reported that the patients gave positive feedback indicating a serious reduction of pain and subjective functional healing after hip or knee arthroplasties; however, they could not find an increase in actual physical activity levels. The current study, however, focuses on whether objective functional capacity increase has any effect on cardiac functions or not instead of the level of actual physical activity. With the exception of SF36 subscale which is the role limitation due to emotional problems, increased HRQOL values were confirmed by the increase in objective capacity measures in the present study. Insufficient physical activity is the most common cause of chronic diseases [7, 19, 20]. It is known that physical activity decreases morbidity and mortality [21-23]. There is no consensus about the amount and intensity of physical activity to help maintain health enhancement; however, it is suggested in different references that 30-minute moderate exercises five days a week, 20-minute vigorous exercise three days a week, or 10000 steps/days can be enough [7, 19, 20, 24]. It has been reported that especially, older adults above 65 years of age cannot reach these activity levels [17]. In the last decades, the increase in older age population has brought out sedentary behavior due to mobilization problems such as osteoarthritis, but in order to maintain health enhancement, it is necessary to provide minimum physical activity levels [20].

However, there is still an ongoing debate concerning the right type, frequency and intensity of physical activity to be used after TKA with no detrimental effect to the implant survival [3, 7, 25, 26]. Cycling, aqua fit, swimming and power walking are favored aerobic activities [25]. It has been also reported that after TKA, patients could not reach aforementioned physical activity levels [6, 7, 18]. Walk was determined to be the optimum type of physical activity in the present study due to the socio-economic facts. A number of previous studies have reported some findings of inverse linear dose-response relationship between physical activity volume and mortality rate [19, 20]. Besides, it has been reported that walking pace is more important in reducing the risk of cardiovascular disease and all-cause mortality risk than walking volume [29]. In order to provide health enhancement, only vigorous activities were recommended by some authors. In contrast, others suggested that moderate activities, such as 10-minute moderate intensity walk are enough [7, 20, 28-30]. Tudor-Locke *et al*. recommended 10-minute moderate intensity walk as a pace of 100 steps/min and completed to 150 minutes per week [30]. These authors proposed 7100 steps/day for physical activity for older adults, indicating physical activity levels of the older adults who have a disability or chronic disease may be less than this [30]. In the present study in order to achieve 150-minute walk per week, the patients were advised to walk in two15-minute sessions of brisk walking, totally 30 min per day. At the end of the first year, brisk walking time was 24.88±13.58 (10-63) minutes/day and 174.23±95.11 (70-441) minutes/week.

With aging, prolongation of early and late mitral inflow velocities (decreasing E/A and Em/Am ratios) and isovolumetric relaxation time take place [11]. It has been suggested that endurance training can prevent deceleration of diastolic filling and myocardial relaxation [8, 11]. Guirado GN *et al*. reported that after 6-months of exercise program, there was no change in left ventricular diastolic functions in older adults [8]. However, it has been reported that with regular and intense physical activity in relatively healthy older adults, left ventricular diastolic functions can be preserved without being affected by aging [9, 11]. In the present, there was a statistically significant increase in E/A and Em/Am ratios in the first year compared to the preoperative echocardiography results, which can be considered as the signs of healing in left ventricular diastolic functions. Left ventricular diastolic functions were evaluated *via* two-dimensional pulsed Doppler echocardiography in the current study. However, in the following studies, more comprehensive examinations, such as three-dimensional echocardiography and left atrium volume measure can be performed to evaluate left ventricular diastolic functions.

This study had some limitations. The accelerometer is reported to be the most accurate objective instrument to measure physical activity [6, 7]. However, we used the pedometer in our study to measure physical activity as it is less costly and easier to use compared to the accelerometer. Besides, the patients with serious mobility problems were excluded due to the nature of this study. Therefore, it might be difficult to compare the current study with others in terms of the findings derived from the patients and objective measures.

## CONCLUSION

After TKA, there was a significant increase in disease-specific, generic evaluations and objective physical capacity measures in the first year and left ventricular diastolic functions may be considered to have recovered in the light of the healing signs *via* echocardiography.

## Figures and Tables

**Fig. (1):**
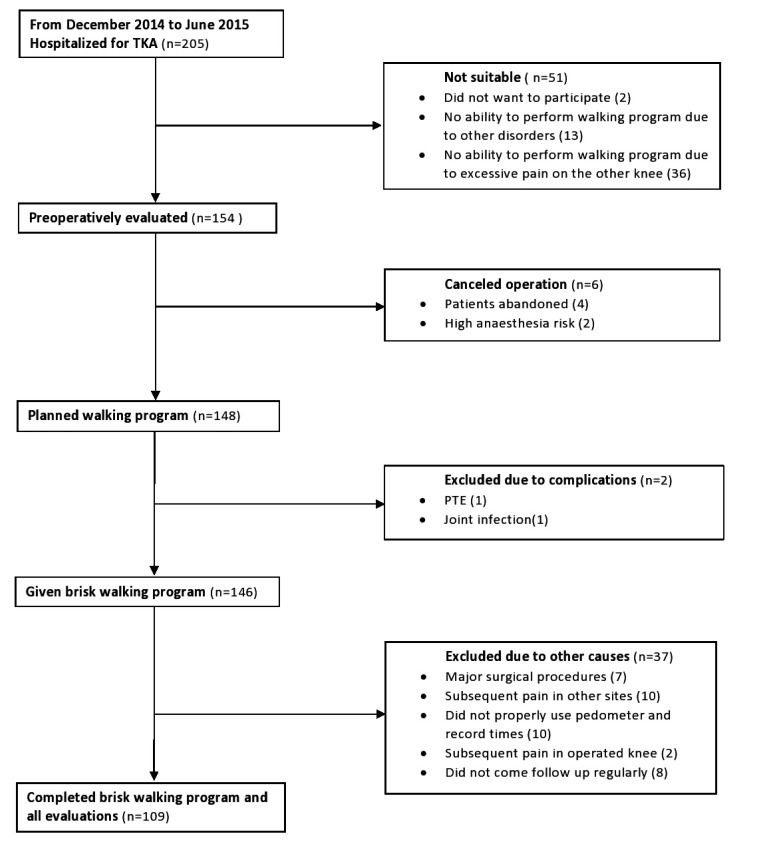
Patient inclusion flow diagram.

**Table 1:** Patients’ characteristics.

**Patients’ characteristics**	
**Number**	109
**Age**	66.75±6.25 (54-85) years-old
**Sex**	96 female. 13 male
**BMI**	31.65±3.95(23.1-41.5) kg/m^2^
**Comorbidities**	55 hypertension 26 diabetes mellitus 9 respiratory (asthma with mild symptoms) 42 other (headache, peptic, treated cancer, bowel diseases, renal diseases relatively mild skeletal disorders)
**Postoperative rehabilitation**	No inpatient Family practitioner, or by oneself
**Living status**	3 patients home alone 106 patients live with family
**Adverse conditions**	2 Haematoma (treated by surgical decompression) 5 wound closure defect (small necrosis in suture line treated *via* dressing) 1 joint infection (treated *via* drainage and debridement, insert exchanging and antibiotics) 2 superficial infection (treated *via* antibiotics) 1 deep vein thrombosis (treated successfully and included in the study) 1 pulmonary thromboembolism (hospitalized in extensive care unit)
**Hospitalization time**	5.44 (4-15) days

**Table 2:** WOMAC scores.

*WOMAC Subscales	**Preoperative**	**Postoperative First Year**	***P*-value**
Pain	17.78±1.66 (14-20)	4.78±2.80 (0-18)	<0.001
Stiffness	4.31±1.6 (1-8)	2.84±0.88 (1-5)	<0.001
Physical function	58.54±3.26(49-66)	21.82±7.82 (6-52)	<0.001

*The WOMAC index [12] is a disease specific measure and includes 24 questions. The questions are grouped into three subscales as pain (20 points), stiffness (8 points), and physical function (68 points). Higher scores demonstrate poor results.

**Table 3:** SF36 survey outcomes.

**SF36 subscales**	**Preoperative**	**Postoperative First Year**	**P value**
PF	27.19±8.89 (10-55)	65.87±9.37 (35-85)	<0.001
BP	8.14±10.33 (0-35)	71.00±16.27 (10-100)	<0.001
RP	2.75±7.86 (0-25)	66.28±22.66 (0-100)	<0.001
RE	77.96±23.06 (0-100)	80.72±21.21 (0-100)	0.11
VT	41.37±13.34 (10-70)	56.10±13.35 (10-85)	<0.001
MH	63.89±17.67 (16-96)	69.74±14.00 (32-96)	<0.001
SF	36.78±15.66 (0-75)	79.59±16.37 (37.50-100)	<0.001
GH	53.89±13.77 (10-85)	59.03±11.04 (15-85)	<0.001

PF: physical functioning, BP: bodily pain, RP: role limitation due to physical problems, RE: role limitation due to emotional problems, VT: energy and vitality, SF: social functioning, GH: general heath perception, MH: mental health, Maximum score of each subscale was 100 points [13].

**Table 4:** Objective physical capacity measures.

**Measures**	**Preoperative**	**Postoperative First Year**	**P-value**
Average step count per day	2199.6±690.8 (453-3800)	4124.3±1638.8 (1700-12096)	<0.001
6MWT (meter)	261.08±65.88 (46-385)	311.92±63.29 (105-422)	<0.001
6MWT (step)	422.65±107.38 (70-615)	502.36±103.16 (180-689)	<0.001
FTSST (sec)	26.73±7.23 (16-56)	22.79±7.59 (13-60)	<0.001

6MWT: six-minute walk test (6MWT): FTSST: the five-times-sit-to-stand test.

**Table 5:** Doppler echocardiographic parameters.

**Parameters**	**Preoperative**	**Postoperative First Year**	**P-value**
LA (mm)	3.62 ±0.29 (3.00-4.50)	3.62±0.32 (2.90-4.40)	0.54
LVEDD (mm)	4.74±0.31(4.00-5.70)	4.72±0.34 (4.00-5.60)	0.36
LVESD (mm)	2.65±0.26 (2.5-2.85)	2.61±0.31(2.40-2.94)	0.42
IVSd (mm)	1.08±0.12 (0.90-1.40	1.09±0.10 (0.90-1.40)	0.17
LVEF (%)	60.23±2.75 (50-65)	60.50±1.92 (55-65)	0.22
E/A	0.71±0.12 (0.40-0.97)	0.77±0.13 (0.48-1.26)	<0.001
Em/Am	0.66±0.13 (0.33-1.22)	0.76± 0.15 (0.41-1.42)	<0.001

LA: left atrium; LVEDD: left ventricular end-diastolic diameter; LVESD: left ventricular end systolic diameter; IVSd: Interventricular septum diameter; LVEF: left ventricular ejection fraction; E/A and Em/Am: early and late mitral inflow velocity ratios.
